# Investigating the Shared Genetic Architecture Between Kidney Function and Alzheimer's Disease Across Ancestries

**DOI:** 10.1002/alz70855_105705

**Published:** 2025-12-24

**Authors:** Diya Yang, Yihe Yang, Xiaofeng Zhu, Nicholas R. Ray, Christiane Reitz, William S Bush

**Affiliations:** ^1^ Case Western Reserve University, Cleveland, OH, USA; ^2^ Columbia University Irving Medical Center, New York, NY, USA

## Abstract

**Background:**

Alzheimer's disease (AD) and kidney function share multiple risk factors and pathogenic mechanisms, particularly through impaired clearance of AD‐related biomarkers. Recent clinical evidence suggests kidney function directly influences AD pathophysiology independent of cardiovascular factors. We aimed to explore this relationship through genetic analyses to minimize confounders like age and lifestyle.

**Method:**

We investigated genome‐wide and local genetic correlations (r_g_) between AD and estimated glomerular filtration rate (eGFR) in European (EUR) and African (AFR) ancestries using LDSC, cond/conjFDR, and LAVA utilizing summary statistics from large‐scale genome‐wide association studies. LAVA measures the strength and direction of local correlations, while cond/conjFDR increases power for pleiotropic variant detection by leveraging conditional false discovery rates.

**Result:**

Genome‐wide correlations between AD and eGFR were nonsignificant (r_g_ values ≈ 0.07) in both ancestries, with regions of strong local correlations observed. In EUR, 52 regions showed significant local correlations (Bonferroni‐corrected), and heritability (AD: h² = 0.05, *p* ≈ 0.025; eGFR: h² = 0.10, *p* < 1E‐10). These regions exhibited balanced bidirectional correlations (26 loci each; positive range: 0.32 to 1.00; negative range: ‐1.00 to ‐0.69), with strongest signals on chr9 (r_g_ = 0.77, *p* = 2.9E−10) and chr5 (r_g_ = −0.66, *p* = 1.34E‐10). The identified loci were linked to genes involved in vascular, cognitive, and inflammatory pathways.

Cond/conjFDR analysis identified 15 loci containing 372 SNPs with significant pleiotropy (conjFDR < 0.05), mapped to genes like *PICALM, SPI1*, and *TOMM40* with previously reported AD associations. In AFR, three loci showed significant negative local correlations (range: ‐0.9 to ‐1), with one AFR‐specific locus on chr11 containing several key genes: *CD81* (inflammation), *STIM1* (calcium signaling and neurodegeneration), *KCNQ1* (potassium channels), and *RRM1* (nephron development and neuronal DNA repair).

**Conclusion:**

Our findings reveal components of shared genetic architecture between AD and kidney function, characterized by a mixture of concordant and discordant associations that are likely driven by specific biological pathways rather than genome‐wide effects. We will focus on refining these associations to determine directionality and causality, identifying key genes driving strong local genetic correlations and their biological pathways, and integrating chronic kidney disease associations alongside eGFR to capture phenotypic variability.

